# Biopatinas on Peperino Stone: Three Eco-Friendly Methods for Their Control and Multi-Technique Approach to Evaluate Their Efficacy

**DOI:** 10.3390/microorganisms13020375

**Published:** 2025-02-08

**Authors:** Daniela Isola, Giuseppe Capobianco, Valery Tovazzi, Claudia Pelosi, Oriana Trotta, Silvia Serranti, Luca Lanteri, Laura Zucconi, Valeria Spizzichino

**Affiliations:** 1 Department of Economics, Engineering, Society and Business Organization (DEIM), University of Tuscia, Largo dell’Università snc, 01100 Viterbo, Italy; valery.tovazzi@gmail.com (V.T.); pelosi@unitus.it (C.P.); llanteri@unitus.it (L.L.); 2 Department of Chemical Engineering, Materials & Environment (DICMA), La Sapienza Rome University, Via Eudossiana 18, 00184 Rome, Italy; oriana.trotta@uniroma1.it (O.T.); silvia.serranti@uniroma1.it (S.S.); 3 Department of Ecological and Biological Sciences (DEB), University of Tuscia, Largo dell’Università snc, 01100 Viterbo, Italy; zucconi@unitus.it; 4 ENEA—Italian National Agency for New Technologies, Energy and Sustainable Economic Development, 00196 Rome, Italy; valeria.spizzichino@enea.it

**Keywords:** BioTersus, black meristematic fungi, DMSO-based gel, hypercolorimetric multispectral imaging, *Knufia*, laser induced fluorescence, Nasier, reflectance spectroradiometry, rock inhabiting fungi

## Abstract

In restoration practice, direct methods become necessary when indirect methods fail and when aesthetic, chemical, or physical biodeteriorative effects threaten the integrity and legibility of the artifact. More effective methods that prioritize the health of workers and the environment are essential for the outdoor stone monument’s conservation. Although several low-impact methods have been proposed, more case studies are needed to address different biopatina types, products, and lithic substrates. Within the COLLINE Project we focused on peperino, a dark volcanic stone widely used in central Italy since the 7th century BCE, because it has been poorly investigated in terms of diversity of biodeteriogens and low-impact methods for their removal. Direct observation, culture methods, and molecular identification have been applied for the identification of biodeteriogens with particular attention to black meristematic fungi. Three low-impact products, namely a dimethyl sulfoxide (DMSO)-based gel, BioTersus^®^ (essential oil-based), and Nasier (enzyme-based) were tested in *ex situ* (on a colonized slab) and *in situ* trials (on the pulpit of the S. Francesco alla Rocca Basilica, Viterbo, Italy). Three analytical methods, namely reflectance spectroradiometry, laser-induced fluorescence (LIF), and hypercolorimetric multispectral imaging (HMI) were used to test the cleaning efficacy. Results evidenced the strong influence of direct irradiation and water availability in the balance and distribution of phototrophs, fungi, and lichens. The low-impact cleaning methods, particularly the DMSO-based gel and BioTersus^®^, effectively remove biodeteriogens from peperino stone while preserving its integrity, offering sustainable solutions for cultural heritage conservation. The instrumental analyses showed that reflectance spectroradiometry and LIF effectively validated the cleaning efficacy, albeit with different sensibility, while HMI, despite spatial constraints, confirmed the three tested cleaning methods do not interfere with peperino stone.

## 1. Introduction

Since the early steps of human history, stone has been the most durable and widely used building material, especially in the Mediterranean basin [1]. The geographical distribution of the natural stones used for monuments reflects local geology, as only precious stones were transported over longer distances [2]. The complexity of Italian geology is reflected in the variety of lithotypes characterizing the territory and, consequently, the materials used by the different Italic populations over the millennia. Among the less known Italian building materials, there is peperino stone. This light, porous, volcanic rock, characterized by light grey to dark grey tones and granular textures, was named after its resemblance to ground pepper, *pepe* in Italian and *piper* in Latin. The term ‘peperino’ has been broadly used in the Italian geological literature, including several different volcanic rocks of the Latium region and central Italy, mainly represented by the phreatomagmatic deposits of the Colli Albani Volcanic District (southeast of Rome), the ignimbrite deposits of the Cimini Mountains near Viterbo (northern Latium, also known as Tuscia), and the Campanian Ignimbrite from the Phlegraean Fields near Naples (the latter is locally known as *piperno*, from the Latin root *piper*) [3]. In upper Latium, this durable stone has been used since the 7th century BCE by the Etruscans and Romans to produce sarcophagi and architectural and ornamental elements (e.g., the Ferento Theatre). In the Middle Ages, entire cities were built using Peperino, including fortified city walls, towers, fountains, and, in Viterbo, also the papal palace. Its use continued though the Renaissance (e.g., Villa Lante, in Bagnaia) and persists to the present day [3].

Any material exposed to outdoor conditions is prone to biotic colonization depending on its macro- and micro-environmental conditions and its bioreceptivity [4,5]. Because of its porosity, any stone can absorb the water necessary to support the growth of both autotrophic and heterotrophic microorganisms, potentially leading to chemical, physical, and aesthetical stone alterations [6,7,8]. Among these, endolithic organisms play a key role in disaggregation processes by growing within stone pores and crevices, cyclically altering their volume in response to water availability [9,10,11,12]. This adaptive strategy, developed by cyanobacteria, algae, fungi, and lichens, gives them a decisive evolutionary advantage by using stone as a refuge and as a shield against external stresses, thereby limiting the effectiveness of treatments [8,13,14]. Traditionally, the prevention and control of biodeteriogen spread are conducted in the early phases of artwork cleaning treatments or after restoration, using biocidal products, to delay recolonization [15,16]. Most of them may contain, at varying concentrations, either alone or in combination, chemicals such as quaternary ammonium compounds, isothiazolinones, octylisothiazolinone, and phenol-based preservatives, among others. Unlike in the past, in the last decades, great attention has been paid to the risks tied to the uncontrolled use of biocides (e.g., Biocidal Products Regulation n. 528/2012/UE) on humans, animals, and the environment [17,18,19]. Many alternative methods, mostly based on essential oils and enzymes, have been proposed and are frequently applied to marbles and different limestones [20,21,22]; scant data are available on peperino stone and its biodeteriogens [23,24]. In this light, within the COLLINE Project supported by the DTC Latium (Center of Excellence named Technological District of Cultural Heritage of Lazio Region), we aimed to contribute to cover this knowledge gap regarding peperino stone. Our goal was to set up, test, and evaluate innovative conservation protocols under real conditions, using integrated diagnostic analysis and sensors for remote monitoring of their effectiveness over time. For the preservation of cultural heritage, the prevention and management of biodeteriogens represent a challenging task, especially due to particularly resistant microorganisms that can further escape potential damage by living below the stone surface. Further methods aimed at controlling or mitigating biodeterioration are needed. Extensive experimentation is necessary to provide restorers greater certainty regarding the efficiency of new methods under various conditions (e.g., different biopatinas and stone types). The direct comparison of products represents a useful tool for evaluating their strengths and limitations. This could lead to an increase in the available reliable protocols and the gradual retirement of the most dangerous traditional biocides, with an emphasis on eco-sustainability. In cultural heritage conservation, instrumental analysis has always been essential for detecting and measuring what the naked eye cannot perceive. As technology advances, more sensitive and versatile instruments are essential. Field comparison of different instruments could guide the development of efficient, customized portable solutions.

This multidisciplinary work, involving biologists, chemists, materials engineers, conservators, and restorers, aims to provide: (i) insight into the biopatinas growing on peperino stone depending on orientation (northern and southeastern sides have been considered), with a focus on phototrophs and rock-inhabiting fungi, these latter well known for their resistance to removal and tolerance to treatments; (ii) an evaluation of their removal using three low-impact methods, namely DMSO-based gel [25,26], BioTersus (an essential oil mix), and Nasier (an enzyme mix); and (iii) a comparison of removal efficiencies by using three analytical methods: reflectance spectroradiometry, laser-induced fluorescence (LIF), and hypercolorimetric multispectral imaging (HMI).

## 2. Materials and Methods

### 2.1. Lithotype Features and Artifact Under Study

The ignimbritic Viterbo peperino (Italian *peperino di Viterbo*) has been considered a thin-grained rock due to the rare presence of 4–5 mm wide crystals [27]. The mineralogy of the rock consists of augite, biotite, and lava, containing fragments of leucite and feldspar; lapilli and zeolitic cement; this last containing minerals in the crystallized or amorphous form, are also present. The texture has been defined as porphyritic, identifying, among the components, quartz, plagioclase, pyroxenes, and glass [28,29,30]. The grayish-brown color is due to femic minerals (ferrous iron and magnesium silicates) and, to a lesser extent, magnetite (Fe_3_O_4_). The physical and mechanical characteristics of this stone improve when extracted at greater depths, with the highest density 2.32∙10^3^ Kg/m^3^ (2.09 are those from the superficial level) and lowest porosity 11.8% (19.9% at surface). The major components of peperino stone are represented by siliceous glass (SiO_2_; 58.16–62.55%), alumina (Al_2_O_3_; 15.45–17.13%), potassium oxide (K_2_O, 5.40–5.75%), and calcium oxide (CaO, 4.27–4.28%) [31].

**Figure 1:**
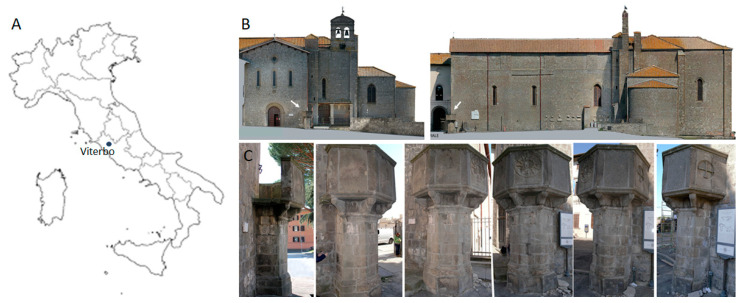
Study site. (**A**) Viterbo on the map of Italy; (**B**) San Francesco alla Rocca Basilica (courtesy of Eagle Project, Rome); (**C**) all sides of the pulpit from the north to the southeast side. The white arrows indicate the pulpit position.

Both *ex situ* and *in situ* experiments were performed. For preliminary *ex situ* comparative tests, a forty-year-old north-exposed colonized peperino slab has been used, while the field study was performed on the pulpit located in the right corner of the San Francesco alla Rocca Basilica (Figure 1) in Viterbo (northern Latium—Central Italy), a Romanesque church whose construction began in 1237 AD. The church incorporated elements of a pre-existing structure, the Palazzo degli Alemanni, which dated back to 1208. It was enlarged with a Gothic transept and later restructured in a Baroque style in 1686. The sacred building is significant for its architectural features, its historical role as a Franciscan theological hub, and for housing notable tombs, including those of Popes Clement IV and Adrian V, both elected in Viterbo during its tenure as the papal seat. After the Allied bombing on 17 January 1944, it was partially destroyed and rebuilt in 1953, restoring the original Romanesque forms, and it received the title of Basilica from Pope Pius XII. The pulpit, with a hexagonal plan, was erected in 1428 in memory of the preaching in Viterbo of Saint Bernardine of Siena (in Italian *San Bernardino da Siena*), as reported in the Latin inscription ‘DIVI BERNARDINI SENENSIS MEMORIA—OB SUAS HIC HABITAS DECLAMATIONES—ASSERVANTUR’. Its major decoration is instead the Sun with twelve rays, representing JHS at the center, the symbol of the Saint [32]. Saint Bernardine was a pivotal preacher of his time, combating corruption, moral decay, and materialism, while defending Catholic orthodoxy.

### 2.2. Innovative Products and Respective Cleaning Protocols

While many low-impact methods have been proposed, more case studies are needed to address various biopatinas, products, and lithic substrates. This is essential to assess the range of effectiveness and promote broader use. Based on previous studies, standardized compositions, and market availability, we selected DMSO-based gel, BioTersus^®^, and Nasier for our experiments. However, in preliminary trials, we compared these chosen products to traditional biocides, which are still commonly used in the field of cultural heritage [33]. In both cases, treatments were applied twice.

#### 2.2.1. DMSO-Based Gel

The DMSO-based gel was prepared following Wolbers’ original recipe [34], by mixing 100 mL of DMSO (VWR Chemicals, BDH, Fontenay-sous-Bois, France) with 10 mL of Ethomeen^®^C25 (CTS, Altavilla Vicentina, Italy) and 3 g of Carbopol^®^934 (Biochim, Milan, Italy) [25,26]. After removing incoherent material by brushing, an overnight water-soaking with hydrophilic cotton patches is required to enhance gel penetration into the lithic matrix. The solvent gel was applied using a wooden spatula, left overnight, and removed as much as possible with dry cotton pads. The surface was then manually brushed and thoroughly rinsed with deionized water. If the result does not meet the visual expectations of the restorer, the protocol can be repeated by applying fresh gel to the still-wet stone.

#### 2.2.2. BioTersus^®^—Essential Oil-Based Product

BioTersus^®^ (Exentiae, Catania, Italy; www.exentiae.it/en/biotersus/, accessed on 10 November 2024) is a biocidal product composed of a mixture of essential oils from *Syzygium aromaticum* (L.) Merr. et Perry (0.5% *v*/*v*), *Thymbra capitata* (L.) Cav. (0.4% *v*/*v*), and *Cinnamomum zeylanicum* Breyne (bark, 0.25% *v*/*v*), having as active molecules carvacrol, cinnamic aldehyde, eugenol, and thymol [35]. This product was first used on the marble statues of the Vatican City State Gardens [36] and then tested on saxicolous lichens [16] and mosaics [37,38]. It is sold as a concentrated solution that requires dilution before use (140 mL to 10 L). Because of its volatility and the need for application on vertical surfaces, the solution was mixed with *Psyllium* seed shells (Greatvita^®^, Hamburg, Germany) as a supporting material (20 g per 300 mL of solution) and left to swell for about 30 min before application to reach the desired consistency. To further reduce evaporation and extend the contact time with the biopatina, the test areas were sealed with cellophane film and plastic tape. The application lasted for 5–7 days. Afterwards, the stone surface was brushed and washed with dH_2_O to remove residues and verify the cleaning effectiveness after each application.

#### 2.2.3. Nasier L01—Enzyme-Based Detergent

Nasier Lapideo L01 (Brenta Chemicals, Lonigo, Italy; https://nasier.it/prodotto/l01-nasier-lapideo/, accessed on 10 November 2024) is a recently patented product designed as a ready-to-use mixture for treating and cleaning colonized stone surfaces. To address operational issues associated with the application of traditional enzymes, this product is thickened and stabilized by micro-nanotechnological particles, eliminating, for example, the need for heating devices before use [39] and refrigeration once opened. Being a patented product, its full composition is not disclosed [40], but trypsin has been accounted at between 0.1 and 0.15%. To date, its application on sandstone and wall paintings has been documented, yielding encouraging results [41,42]. The supplier recommends applications lasting 30–45 min. We applied the highest suggested resting time.

### 2.3. Ex Situ Comparative Experiment

Preliminary tests were performed on a forty-year-old, north exposed, colonized peperino slab. It was divided into six squared sectors, each approximately 5 cm per side, using paper tape. Each area was subjected to a different treatment, while the surrounding area was left untreated as a negative control. The selected traditional biocides were Preventol^®^ RI80 (PREV; CTS, Altavilla Vicentina, Italy), Biotin T (BioT; CTS, Altavilla Vicentina, Italy), and Biotin R (BioR; CTS, Altavilla Vicentina, Italy). Biocides were applied as water solutions (except BioR diluted in white spirit). The highest concentrations recommended by the supplier were used: 2% for PREV, 3% for BioT, and 5% for BioR. The active compounds varied significantly among the products, being unique for PREV (benzalkonium chloride 80%), while the others contain multiple multitarget components. Indeed, BioT is composed of didecyl-dimethyl ammonium chloride (40.0–60.0%), N-octyl-isothiazolinone (7.0–10.0%), formic acid (2.0–2.5%), and isopropyl alcohol (15.0–20.0%) [43], while BioR contains 3-iodo-2-propynyl butylcarbamate (10–25%) and 2-n-octyl-4-isothiazolin-3-one (3–5%) dissolved in 2-(2-butoxyethoxy) ethanol [44]. Traditional biocides were applied by brush tapping on the surface until refusal (i.e., saturation). This method was chosen to minimize operator risk and increase product penetration. The remaining squares were treated with BioTersus at 1.4% (as recommended by the supplier), Nasier, and a DMSO-based gel. The traditional biocides and BioTersus were applied, sealed with plastic foil and tape, and left to rest for a week. Nasier was left to act for 45 min, and the DMSO-based gel was left overnight, according to their respective protocols. All the treatments were applied twice. A USB portable light microscope (DigiMicro Profi, DNT Innovations GmbH, Leer, Germany) and reference color chart and image color curve analysis (e.g., RGB and Luminosity) performed using Photoshop^®^ v. CC 2021 [26] were used to verify any unwanted color change of the stone.

### 2.4. In Situ Experiment

**Figure 2:**
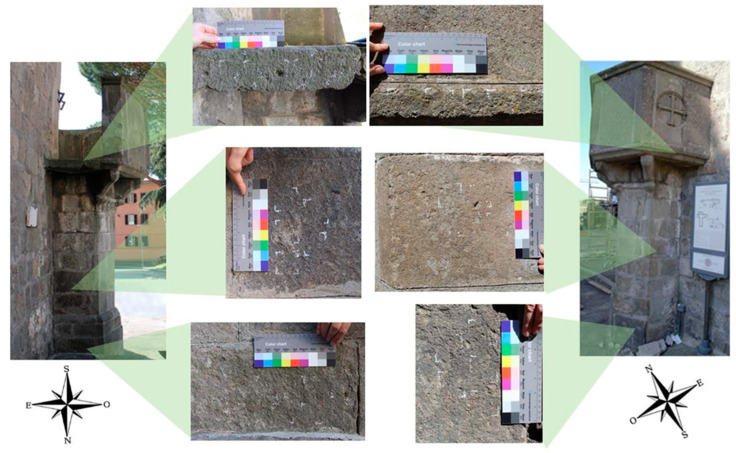
*In situ* experiment test areas. To have the broader representation of biopatinas, the pulpit’s sides exposed to the north (left) and southeast (right) were studied. Per each side were considered three different heights from the ground (namely high, middle, and low positions).

Due to its hexagonal plan, the pulpit has different exposure on each side (Figure 1). Since exposure and water availability strongly affect colonization phenomena, both the north (N) and southeast (SE) facing sides were considered, with three elevations on each site from the ground: high (H), middle (M), and low (L) positions (Figure 2). Therefore, the test sites were identified as NH, NM, and NL for the north exposed side and SEH, SEM, and SEL for the southeast facing side of the pulpit. Since there were three planned treatments, a total of nine test square areas, each measuring 4.5 cm per side, were marked on each side, resulting in a total of eighteen test areas. In addition to the three treatment areas per site, two additional squares were designated as negative controls, except at the NH site due to space constraints. The treatments (i.e., DMSO-based gel, BioTersus, and Nasier L01) were applied twice.

### 2.5. Physical Measurements

#### 2.5.1. Reflectance Spectroradiometry

The spectral measurements were performed using an ASD FieldSpec^®^ 4 Standard-Res Spectroradiometer (Malvern Panalytical, Malvern, UK), covering a spectral range from 350 to 2500 nm. The instrument offers a sampling bandwidth of 1.4 nm in the 350–1000 nm range and 2 nm in the 1000–2500 nm range. The spectral resolution was 3 nm at 700 nm, 10 nm at 1400 nm, and 10 nm at 2100 nm. The measurements were conducted using a high-intensity contact probe (Analytical Spectral Devices) equipped with a halogen light source (2900 ± 10% K). The contact probe provided a spot size with a 10 mm diameter, ensuring accurate targeting of the samples. For each measurement area, 40 spectra were collected per analysis square, covering an area of 5 cm^2^. The reference dataset consisted of 40 spectra from the biological patina and 40 spectra from the peperino reference sample, ensuring comprehensive coverage of the targeted areas. In the northern portion of the pulpit, 120 spectra were collected from the upper side, 160 from the middle side, and 160 from the lower side. These measurements were repeated after cleaning treatment, resulting in a total of 1320 spectra acquired. Similarly, in the southeastern portion of the pulpit, 160 spectra were collected from each of the upper, middle, and lower sides, also repeated after cleaning and treatment, yielding a total of 1440 spectra. This extensive dataset provided a robust foundation for building a PCA-LDA classification model and monitoring the presence of biological patinas. PCA was used to identify the main sources of variation, while LDA was applied to achieve class separation (biological patina vs. peperino). Model robustness was confirmed through 10-fold cross-validation, minimizing overfitting and ensuring reliable results.

To enhance the detection of biological patina, we focused on the spectral region from 400 to 1000 nm, allowing for targeted preprocessing and data reduction. Specifically, principal component analysis (PCA) further emphasized the region between 500 and 700 nm due to the presence of photosynthetic pigments such as chlorophyll.

##### Multivariate Data Analysis on Reflectance Data

The acquired spectral data were analyzed using PLS_Toolbox (Version 9.3.1 Eigenvector Research, Inc.) for the preprocessing algorithm and principal component analysis (PCA). The Statistics and Machine Learning Toolbox™ was used to develop linear discriminant analysis (LDA). All data were processed within the MATLAB^®^ environment (R2024b, The Mathworks, Inc., Natick, MA, USA).

##### Preprocessing

Preprocessing of VIS-NIR (near-infrared) data is a crucial step in spectral analysis to reduce variability due to instrumental noise, variations in illumination, or physical effects of the sample, like light scattering, which can affect the accuracy and reliability of the analysis [45]. To improve signal quality in this study, the following preprocessors were used:

Detrend: This method removes linear trends in the spectral data, such as systematic changes in the baseline. Linear variations can be caused by external factors, such as sample geometry or gradual changes in measurement conditions. Removing these trends is essential for analyses where the baseline directly affects the accurate detection of spectral absorption [46].

Derivative (Savitzky–Golay): The calculation of the first derivative of spectral data emphasizes changes in the shape of the signal, removing global offsets and trends. The Savitzky–Golay filter is used to improve the resolution of spectral bands, reducing the influence of global variation while preserving the integrity of the signal by smoothing the noise [45,47].

Absolute Value: This preprocessor transforms negative values, which may appear after applying derivatives, into positive values, simplifying the interpretation and subsequent analysis of the data, especially in contexts where negative values are irrelevant to the physical analysis.

Normalization: This operation scales the data so that the maximum value in each spectrum is set to 1. This reduces variability caused by differences in absolute intensity between samples, considering that the analyzed acquisitions were performed on different days and under varying solar lighting conditions, which can contribute to noise. Normalization thus makes the data more comparable across samples [48].

Mean Center: This method centers the data by subtracting the mean of each variable from its corresponding values, centering the data around zero. Mean centering is useful in multivariate analysis because it highlights only significant variations in the data, removing systematic differences between samples and facilitating more precise interpretation of latent structures [49].

The following preprocessing sequence was applied to the VIS-NIR data: the spectra were initially treated with ‘Detrend’ (Linear) and ‘Normalize’ (inf-Norm, Maximum = 1) to reduce variability related to light scattering caused by the porous texture of the peperino stone. Subsequently, a ‘1st Derivative’ using the Savitzky–Golay filter (order: 2, window: 21 points, incl only, tails: weighted) was applied to enhance spectral features associated with the biological patina, focusing on variations around 700 nm, which are indicative of chlorophyll a. To improve comparability among the processed signals, the ‘Absolute Value’ was used. Finally, before performing PCA, the data were mean-centered (Mean Center) to facilitate the identification of latent variations among the samples.

##### Principal Component Analysis (PCA)

PCA is a statistical technique used to explore data and reduce the dimensionality of large datasets while preserving as much variability as possible. It works by transforming the original variables into a new set of uncorrelated variables, called principal components, which are ordered by the amount of variance they explain. The first principal component captures the most variance, and each subsequent component captures the maximum remaining variance subject to the constraint of being orthogonal to the previous components. PCA helps to simplify data analysis, identify patterns, classify samples, and reduce noise, making it particularly useful in chemometric approaches for analyzing complex datasets [50]. In this study, PCA was used as a data reduction technique to filter out irrelevant information, allowing the analysis to focus exclusively on major spectral contributions associated with variations in the biological patina compared to the peperino stone.

##### PCA-LDA for Classifying and Estimating Biological Patina

Linear Discriminant Analysis (LDA) is a supervised statistical technique used for both dimensionality reduction and classification in datasets with predefined classes. It is often applied after Principal Component Analysis (PCA), leveraging the reduced dimensionality of the data while enhancing class separability. While PCA focuses on preserving variance without considering class labels, LDA aims to maximize separability between classes by projecting the data onto a lower-dimensional space. This approach benefits from the noise reduction and simplification provided by PCA, allowing LDA to more effectively distinguish between categories. LDA achieves this by finding linear combinations of features that best separate the classes, maximized the distance between class means while minimizing the spread within each class. LDA is particularly effective in scenarios where the assumption of normally distributed classes and equal covariance among them holds true, making it widely used in fields such as pattern recognition, face recognition, and chemometrics, where distinguishing between different categories is essential for accurate data interpretation [51]. To ensure the robustness of the model, a cross-validation approach was employed using a k10-fold discriminant analysis [52]. This approach utilized two predictor variables, X1 and X2, to classify the response variable Y, based on a total of 80 observations. The cross-validation was executed by partitioning the dataset into 10 subsets, allowing each subset to serve as a validation set while the remaining subsets were used for training. This method not only helps in assessing the predictive performance of the model, but also aids in minimizing overfitting. The classes being predicted were ‘Biopatinas’ and ‘Peperino stone’. To estimate the biological patina and the peperino in the measurement areas before and after the treatments, predictions from the LDA model for each measurement point obtained were used. The means of the populations and their respective percentages for both classes were then calculated for each measurement and/or treatment area. Finally, the obtained results before and after the treatment were compared using a summary graph.

#### 2.5.2. Laser-Induced Fluorescence (LIF)

LIF is a powerful real-time remote diagnostic tool that has been successfully applied to historical artworks, enabling the detection of pigments, biological attacks, and traces of previous restorations [53,54,55]. It is based on the collection of the fluorescence signal produced by the target under study after an excitation by laser radiation, typically in the UV range. For the characterization of treated areas, a prototype designed and built up at the Diagnostic and Metrology Laboratory of the ENEA research center of Frascati was used. Called CALIFFO (Compact Advanced Laser Induced Fluorescence Friendly Operating system), it is particularly designed to on-site measurements due to its compact, lightweight design. The device is completely wireless and remotely controlled via Bluetooth or Wi-Fi using any mobile device. It operates at a one-meter distance from the surface target, avoiding, then, any contact. This feature also allows for operating on targets barely accessible or protected (for example by glass or gratings). Its main field of application is the recognition of biological pigments (mainly chlorophyll-*a*) and monitoring of bio-attacks on historical and artistic surfaces due to the use of a continuous laser emitting at 405 nm, a wavelength very close to an absorption maximum of the chlorophyll-*a* (Chl *a*) pigment, with low divergence (0.6 mrad) and high optical output power (100 mW). An Ocean Optics USB4000 is used as a spectrometer, in the range 337–751 nm, with a spectral resolution of 0.5 nm. Mechanical frames were realized with a 3D laser printer [56]. The system was used to carry out 10 × 10 pixel scans on every studied area. The outputs are fluorescence spectra of each single point. Processing these data, it is possible to obtain mean fluorescence spectra of the whole squared areas, as well as maps highlighting specific materials and forms of degradation.

##### LIF Data Processing

For each spectrum obtained, the intensity was normalized to the integrated signal over the considered spectral range (i.e., 407–800 nm) to ensure the spectra were independent of experimental fluctuations (e.g., laser power, geometry) and comparable among them. Fluorescence maps were then been built up at 440, 489, 511, 550, 603, 680, 740, and 780 nm. These wavelengths were selected for their relevance in characterizing the studied stone surfaces. For instance, Chl *a* exhibits two fluorescence emission maxima at about 680 and 720–740 nm [57,58], while many stones have local maxima in the region of 400–550 nm (mainly metamorphic and sedimentary stones) or in the range of 750–800 nm (magmatic stones) [59]. Finally, additional maps were generated to highlight specific substances and materials. To detect the possible presence of Chl *a*, the ratio (I_680_ + I_740_)/I_550_ was calculated for each pixel and used to create distribution maps.

#### 2.5.3. Hypercolorimetric Multispectral Imaging (HMI)

This non-invasive diagnostic method enables quick, precise, and repeatable *in situ* spectral reflectance measurements, ranging from ultraviolet (UV) to near-infrared, for detailed surface analysis. The system was developed by Profilocolore (Rome, Italy) with the aim of transforming a commercial digital camera into a radiometric instrument of measurement [60]. The HMI technique uses a modified digital Nikon D800 camera (Nital SpA Moncalieri Torino, Italy) designed to cover the full spectral range from 300 to 1000 nm. The illumination system consists of two modified flashes covering the above-indicated spectral range. The acquisition setup includes two filters, named A and B, covering the UV-Vis and Vis-NIR regions, respectively, whose spectra have been previously published [61]. To produce radiometrically and colorimetrically calibrated images, white patches and a color-checker (36 color samples from the Natural Color System^®^ ©, NCS catalog) were positioned in the scene around the selected stone surfaces. The need to position the reference whites and the color checker restricted the acquisition to the median test areas of the selected surfaces. Specifically, the median area on the southeast side (SEM) and on the north side (NM) were acquired.

The HMI system is completed by two software programs, SpectraPick^®^ (v1.1, created by Profilocolore, Rome, Italy) and PickViewer^®^ (v1.0, created by Profilocolore). The first software is used for the most important step of the procedure, i.e., the calibration, which starts from the two acquired images (one with the filer A and the other with the filter B) to produce seven monochromatic images centered at 350, 450, 550, 650, 750, 850, and 950 nm, and the RGB output, all contained in a single folder. The other software, PickViewer^®^, is used to process the calibrated images to obtain different kinds of information about color, spectral reflectance, similarity maps, principal component analysis, etc. In the present study, spectral similarity mapping and spectral reflectance comparison were used to compare surface characteristics before and after the treatments and to identify differences caused by the treatments and the different products used. These analyses applied PickViewer^®^ algorithms on 39 × 39 pixel areas.

The potentiality of the HMI system has been widely demonstrated, especially in the analysis of paintings, but it has never been applied to stone surfaces before [42,61,62].

### 2.6. Sampling and Biopatina Characterization

Biological samples were collected using sterile cotton swabs from each test area in both *ex situ* and *in situ* experiments before the treatments to characterize the biopatinas through the identification of dominant species. The sampling was repeated seven months later to allow any remaining organisms time to recover from potential minor damage caused by the treatment. The samples were suspended in 1 mL of sterile saline solution (NaCl 0.9%), plated at various dilutions onto Dichloran Rose Bengal Chloramphenicol agar (DRBC, VWR) and Malt agar (MA, malt extract 30 g/L, bacteriological agar 15 g/L; VWR) plates, and incubated at 15 °C for about 2 months. All these conditions were applied to favor the isolation of black fungi, which are known to be slow-growing representatives of lithobiontic biofilms. Additionally, 100 μL aliquots from the suspensions were used to inoculate flasks containing 10 mL of BlueGreen-11 freshwater medium (BG11, Sigma Aldrich, St. Louis, MO, USA) for cyanobacteria and flasks containing 10 mL of Bold Basal Medium (BBM, Sigma Aldrich) for micro-algae, according to the guidelines outlined in NORMAL 9/88 [63]. Cyanobacterial and algal cultures were maintained for 6 months under cool-white fluorescent illumination (Osram Dulux L 36W/840 Lumilux, 2900 lumens), with a 12 h photoperiod at room temperature (20 ± 1 °C) [64].

Although eubacteria can play an important role in biofilm formation and stone material degradation, we focused on black fungi (BF) because they are considered suitable reference organisms for compounds testing [26,65]. Pure black fungal colonies were isolated on MA plates and identified by molecular methods using ITS4 and ITS5 primers [66]. The amplicons sequences were compared to the GenBank database using BLASTn. The interest for the phototrophic fraction is instead tied to their ability to promote biofilm formation and rapidly produce disfiguring color changes. No isolation on solid media was performed. Morphological identification of the phototrophic fraction was carried out by preparing microscope slides from liquid enrichments and using the analytical keys provided by Guiry and Guiry [67]. Lichens were identified using identification keys in ITALIC 7.0 [68].

After the treatments and instrumental analyses, culture tests were repeated from the treated areas to assess short-term microbial re-growth. All isolated fungal strains are preserved in the Culture Collection of Fungi from Extreme Environments (CCFEE, Viterbo, Italy).

### 2.7. Experiment Synopsis

**Figure 3:**
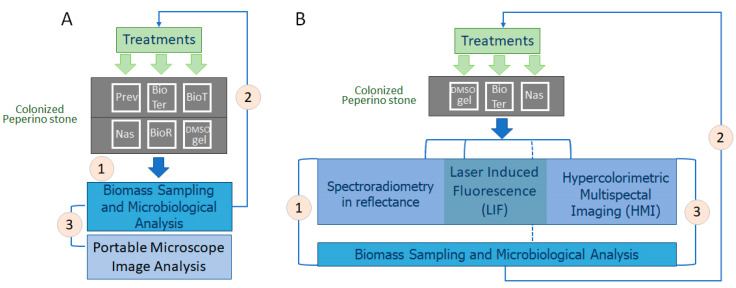
*Ex situ* and *in situ* experiment synopsis. (**A**) In the *ex situ* experiment, the colonized peperino stone was 1. subjected to biomass sampling and microbiological analysis; 2. treated with Preventol RI80 3%, BioTersus^®^ (BioTer), Biotin T 3% (BioT), Nasier L01 (Nas), Biotin R 5% (BioR), and a DMSO-based gel (DMSO gel); and 3. observed using a portable microscope to verify color changes and sampled to verify the efficiency of biopatina removal. (**B**) In the *in situ* experiment, the colonized peperino stone was 1. subjected to full range spectroradiometry in reflectance, LIF, and HMI measurements; the sampled biomass was then analyzed for microbiological components; 2. treated using a DMSO-based gel, BioTersus^®^, and Nasier L01; 3. re-subjected to full range spectroradiometry in reflectance, LIF, and HMI measurements followed by microbiological analysis to verify the efficiency of biopatina removal. For technical aspects, as explained in Section 2.5.3, HMI measurements were performed on the median test areas only, while biomass sampling always followed instrumental analysis.

In Figure 3, the resuming scheme of the steps composing *ex situ* and *in situ* experiments is reported. In both experiments, biomass sampling was conducted three months after the treatments. In the *in situ* experiment, instrumental measurements were taken before the sampling. Pictures were taken over the seven months following the treatments.

## 3. Results

### 3.1. Ex Situ Comparative Experiment

**Figure 4:**
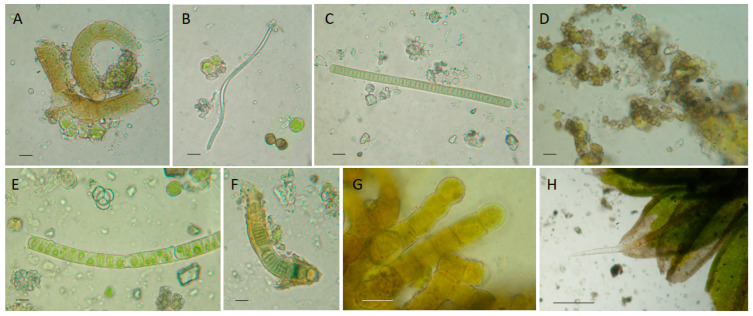
Peperino slab biodeteriogens. (**A**) *Calothrix parietina* Thuret ex Bornet & Flahault; (**B**) *Leptolyngbya* sp.; (**C**) *Lyngbya* sp.; (**D**) Black meristematic fungi; (**E**) *Klebsormidium flaccidum* (Kützing) P.C. Silva, Mattox & W.H.Blackwell; (**F**) *Calothrix parietina;* (**G**) *Trentepohlia* sp.; (**H**) *Tortula muralis* Hedw. Scale bars (**A**–**G**), 10 μm; (**H**), 200 μm.

The peperino slab was colonized (Figure 4) by cyanobacteria (e.g., *Calothrix parietina* Thuret ex Bornet & Flahault, *Leptolyngbya* sp., *Lyngbya* sp.), algae (e.g., *Klebsormidium flaccidum* (Kützing) P.C. Silva, Mattox & W.H. Blackwell, *Trentepohlia* sp.), and moss patches (*Tortula muralis* Hedw.). Black fungi were observed, either alone or in contiguity to phototrophs. The isolated BF strain shares 98% similarity with *Coniosporium uncinatum* CCFEE 5820 (Table S1).

**Figure 5:**
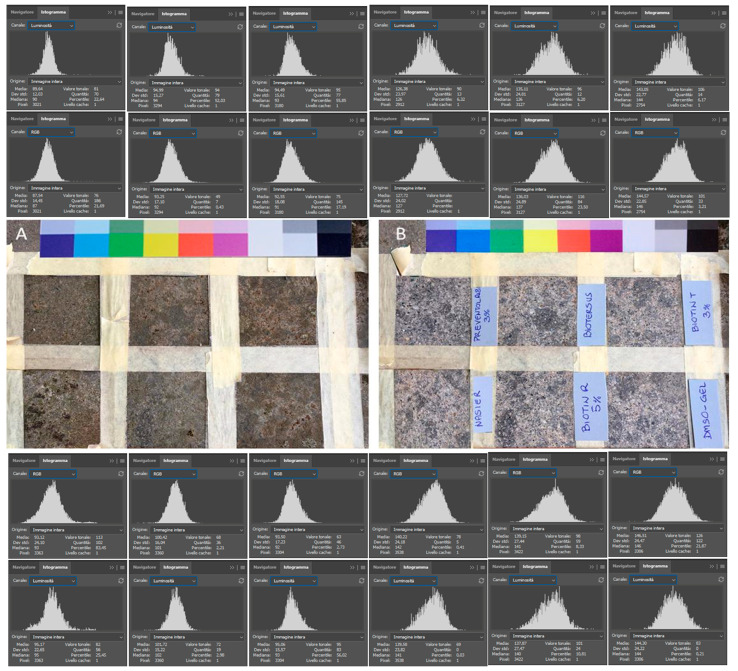
*Ex situ* comparative test. (**A**) the colonized north-exposed peperino slab before treatments, and (**B**) treated peperino slab after two applications each. In correspondence to each test area are reported the color curves (i.e., RGB and Luminosity) achieved using Photoshop^®^ CC 2021.

All the applied methods lead to complete removal of the biological patina (Figure 5), with no side effects recorded. No relevant differences have been recorded among traditional and innovative cleaning methods. In general, after treatments, increases in average luminosity were always recorded, with values ranging from 36.74 (Prev80) to 49.24 (DMSO-gel). A broadening of the histogram spikes was also recorded, most probably related to the increased visibility of dark and light grains composing the peperino stone.

### 3.2. In Situ Experiment: Biological Patina Composition and Removal Yields

#### 3.2.1. Biological Patina Composition

**Figure 6:**
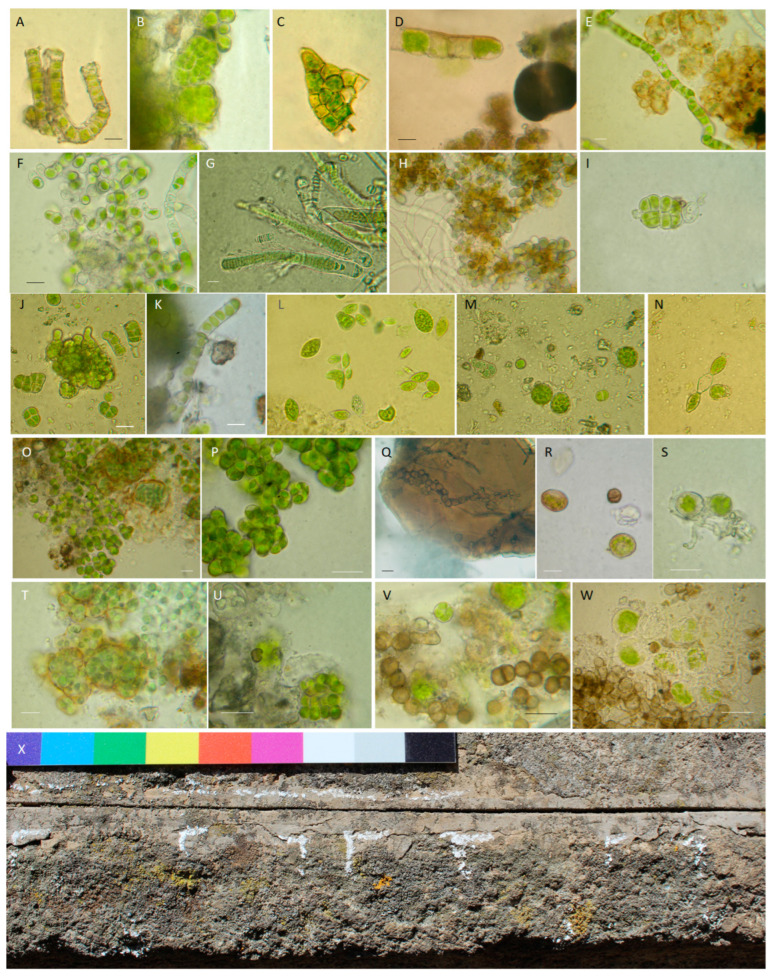
Overview of the biodeteriogens found on the San Francesco alla Rocca pulpit. (**A**–**I**) North-facing side, upper part. (**A**) *Klebsormidium flaccidum*; (**B**) *Apatococcus lobatus* (Chodat) J.B. Petersen; (**C**) *Pleurocapsa* sp.; (**D**–**F**) *Kl. flaccidum; (***G**) *Calothrix parietina;* (**H**) *Klebsormidium* sp.; (**I**) *Chroococcus turgidus* (Kützing) Nägeli. (**J**–**N**) North-facing side, lower part. (**J**) *Pleurocapsa* sp.; (**K**) *Kl. flaccidum*; (**L**) *Oocystis* sp. and *Euglena splendens* P.A. Dangeard; (**M**) *Chlorogloea* sp., (**N**) *Euglena* sp. (**O**–**X**) Southeast-facing side, upper part. (**O**,**P**) *Apatococcus lobatus* (Chodat) J.B. Petersen; (**Q**) black meristematic fungi; (**R**) *Gloeocapsa novacekii* Komárek & Anagnotidis; (**S**) unconventional lichenic structures; (**T**) *Chlorogloea* sp.; (**U**) *Apatococcus lobatus*; (**V**,**W**) possible interactions between black fungi and phototrophs. (**X**) *Acarospora fuscata* (Schrad.) Arnold; *Candelariella vitellina* (Hoffm.) Müll. Arg., *Xanthoria parietina* (L.) Th. Fr., and *Scoliciosporum umbrinum* (Ach.) Arnold. Scale bars, 10 μm.

Direct observations of biological colonization and culture-based analysis allowed us to qualitatively and quantitatively distinguish differences between north- and south-east-exposed areas (Table 1). Biopatinas on the northern side of the pulpit were predominantly composed of phototrophic organisms. The biofilm thickness decreased in order from the upper to the lower and median parts. The species and genera found were *Apatococcus lobatus, Chroococcus turgidus*, *Calothrix parietina*, *Ulothrix* sp., *Desmococcus* sp., *Klebsormidium flaccidum*, and *Klebsormidium* sp. in the upper part (Figure 6A–I), *Oocystis* sp., *Pleurocapsa* sp., and *Scenedesmus* sp. in the lower part (Figure 6J–N), and occasionally *Klebsormidium* sp. in the middle part (very rare, about a record each for 25 microscope-explored fields). On the northern side, BFs showing the closest identity with *Knufia marmoricola* CCFEE 5886 (KP791790, 99.83%) and *Knufia petricola* IHEM:22814 (OW986671, 99.83%) were isolated from the median part, as well as strains sharing identity scores with *Setophaeosphaeria badalingensis* MK311292 (99.28%) and *Scolecobasidium robustum* NR_155567 (96.52%) from the lower part. Table S1 reports the accession numbers of the new fungal isolates.

Colonization on the southeastern side was limited to the upper part, where lichens, phototrophs, and BFs were frequent (Figure 6O–X). Lichens such as *Acarospora fuscata* (Schrad.) Arnold, *Candelariella vitellina* (Hoffm.) Müll. Arg., *Xanthoria parietina* (L.) Th. Fr., and *Scoliciosporum umbrinum* (Arch.) Arnold were found (Figure 6X). The phototrophic component was diversified (e.g., *Apatococcus lobatus*, *Gloeocapsa novacekii*, and *Chlorogloea* sp.) and in part associated into unconventional lichenic structures. BF diversity was visibly higher, with records close to *Knufia marmoricola*, *Coniosporium uncinatum*, and several unknown species (Table 1 and Table S1) belonging to the Eurotiomycetes (CCFEE 10049, 10052) and Dothideomycetes (CCFEE 10050, 10051, 10053) classes.

**Table 1:** Main organisms composing the sampled biopatinas. Complete BLAST best match data are reported in Table S1.

	*Ex situ*	*In situ*		
Sampled Area	Peperino Slab	Pulpit, North (N)	Pulpit, Southeast (SE)	Organisms
Slab	*Tortula muralis*			Mosses
	*Calothrix parietina* *Leptolyngbya* sp. *Lyngbya* sp.			Cyanobacteria
	*Klebsormidium flaccidum Trentepohlia* sp.			Algae
	*Coniosporium* sp.			Fungi
High (H)		*Calothrix parietina* *Chroococcus turgidus* *Pleurocapsa* sp.	*Chlorogloea* sp. *Gloeocapsa novacekii*	Cyanobacteria
		*Apatococcus lobatus* *Klebsormidium flaccidum Klebsormudium* sp.	*Apatococcus lobatus*	Algae
		*Setosphaeosphaeria* sp	*Knufia marmoricola Coniosporium uncinatum Constantinomyces* sp. *Exophiala* sp. Dothideomycetes sp. Herpotrichiellaceae sp.	Fungi
		/	*Acarospora fuscata* *Candelariella vitellina* *Xanthoria parietina* *Scoliciosporum umbrinum*	Lichens
Middle (M)		/	None	Cyanobacteria
		*Klebsormidium* sp.		Algae
		*Knufia marmoricola* *Knufia petricola*		Fungi
Low (L)		*Chlorogloea* sp. *Pleurocapsa* sp.	None	Cyanobacteria
		*Euglena* sp. * Euglena splendens * Klebsormidium flaccidum *Oocystis* sp.		Algae
		*Scolecobasidium* sp. *Setosphaeosphaeria* sp.		Fungi

‘None’ means that no biological patina has been recorded. ‘/’ indicates the absence of that category of biodeteriogens. * *Euglena* species traditionally considered algae belong instead to Discoba lineage (Euglenozoa). Grey areas are used to improve readability.

#### 3.2.2. Field Practical Evidence on the Application and Removal of Treatments

**Figure 7:**
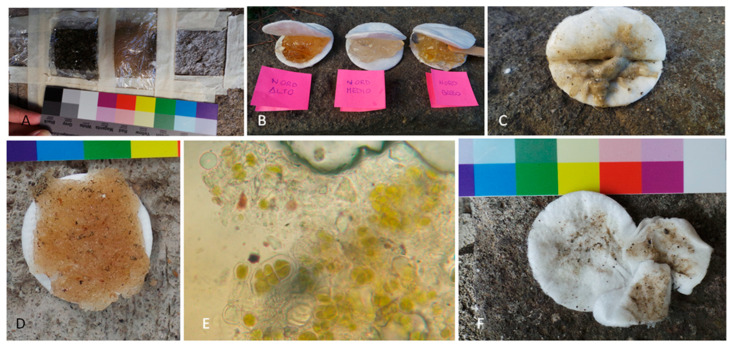
Application notes. (**A**) The three treatments applied, the third (whitish) being enzyme-based (Nasier). (**B**) Appearance of the removed DMSO-based gel after overnight application on the northern site (shown in order of high, middle, and low). (**C**) Washing out of the DMSO-based gel. (**D**) BioTersus *Psyllium* pad from NL. (**E**) Microscope observation of BioTersus *Psyllium* pads showing the color change that occurred in phototrophic organisms compared to the brilliant green observed in untreated phototrophs (see Figure 6J–N). (**F**) Cotton pads from Nasier SEH washing out.

The DMSO-based gel, initially faintly yellowish and transparent upon application (a favorable factor, as it allows to observe the action of the product on the surface), darkened proportionally with the thickness of the phototrophic biofilm component (Figure 7B). Additional dirt was removed during washing and brushing thanks to the fine-bubbled texture of the remaining gel (Figure 7C).

The BioTersus poultice has a strong, albeit pleasant scent, even when used in open air. The *Psyllium* seed shells can serve as a suitable supporting material due to their soaking and adhering properties (Figure 7D), allowing one to record color alterations on photosynthetic organisms with an optical microscope (Figure 7E). Moreover, adhering properties can be a drawback if the poultice dehydrates during the application, and, in consideration of this possible tearing action, it is imperative to consider the condition of the substrate, evaluating a possible pre-consolidation treatment in case of decohesion phenomena. For these reasons, the poultice strength should be customized based on the application duration, ambient temperatures, and the orientation of the surface to be treated.

The vertical walls and peperino porosity result in conditions that require attention during Nasier application [42]. Indeed, the application took more time than expected because it was necessary to redistribute the poured material onto the overlying surface. Its removal could be performed through mechanical action and abundant rinsing, during which time fine bubbles are formed. The mechanical action would be stronger if the enzyme layer dehydrates during treatment standing (Figure 7A). A limitation of this technique is the environmental conditions, as the enzymes can only be applied within a certain temperature range, outside of which they cannot perform their function. Furthermore, the necessity of thoroughly rinsing the surface recalls the cohesion problem underlined for the BioTersus treatment.

#### 3.2.3. Biopatina Removal Yields

**Figure 8:**
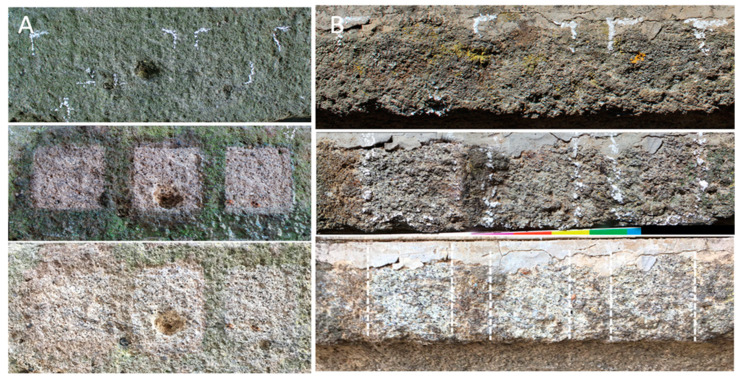
Biopatina removal yields using, in order, DMSO-based gel, BioTersus, and Nasier. (**A**) NH untreated phototroph-dominated biopatina followed by the appearance of the same area at the end of the cleaning procedure (4 October 2022) and after seven months (7 May 2023); (**B**) SHE untreated biopatina where lichens such as *Acarospora fuscata* (Schrad.) Arnold, *Candelariella vitellina* (Hoffm.) Müll. Arg., *Xanthoria parietina* (L.) Th. Fr., and *Scoliciosporum umbrinum* (Arch.) Arnold were visible. Below is the same area at end of the cleaning procedure (4 October 2022) and after seven months (7 May 2023). The dashed lines are superimposed on the figure to facilitate a quick comparison with the images above.

The most evident effects of treatments were appreciable both in the NH and SEH sites (Figure 8). After the two treatments runs in NH, all three applied products completely removed the biofilm. Seven months later (on 7 May 2023), no growth was detected from cultures of the treated areas. Additionally, a clear halo was observed around the tiles treated with the DMSO gel and BioTersus (highlighted in Figure S1B), which overlaps with the area affected by some possible residue of wash up (Figure S1A).

The removal of the lichen-dominated biofilm characterizing the SEH area was different. The highest removal was achieved with the DMSO-based gel, followed by BioTersus, having some relict lichens in the upper part and on the left side. In contrast, Nasier resulted in unsatisfactory lichen removal under the tested conditions. After seven months, no growth was recorded by culture.

In the other colonized areas (namely NM and NL), the cleaning results were not so evident.

### 3.3. In Situ Experiment: Instrumental Measurements Before and After Treatments

#### 3.3.1. Reflectance Spectroradiometry

To analyze the spectral characteristics in the VIS-NIR range on the pulpit, the reference area chosen was the stone platform of the pulpit, covered by uniform colonization (Figure S2A). A 5 × 5 × 2 cm peperino stone sample without biological patina served as a reference (Figure S2B). The analysis of the reflectance spectra (Figure S2C) for the reference peperino stone sample (green line) and the biological patina (red line) reveals significant differences in the 500–700 nm region. Specifically, the biological patina spectrum shows an initial inflection around 500 nm, a second at 580 nm, and a prominent change near 700 nm, which indicates absorption by photosynthetic pigments, such as chlorophyll. To enhance these spectral features and reduce variability related to light scattering, a preprocessing workflow including ‘Detrend’, ‘1st Derivative’, and ‘Normalization’ was applied. The resulting spectra, shown in Figure S2D, highlight the improved focus on relevant spectral variations. PCA showed 97.77% cumulative variance with two principal components, clearly separating the peperino and patina samples (Figure S2E). The loadings plot (Figure S2F) revealed that PC1 distinguishes between the samples based on spectral contributions around 500–550 nm and 700 nm, while PC2 captures variability from 400–550 nm and 800–1000 nm. Starting from the PCA model developed on the reference samples, data reduction was performed by selecting the principal components that best represented the variance in the data (i.e., 2 PCs). This approach simplified the dataset by retaining the most significant information while reducing noise and irrelevant features. The score plot generated from the reference samples served as a constraint for the test sets, ensuring that the same principal components were applied to the unknown data. Based on the PCA scores from this constrained model, an LDA classification model was constructed. The model was trained and validated through calibration and cross-validation, demonstrating perfect discrimination between the biological patina and the peperino stone samples. The effectiveness of this classification is shown in the confusion matrices in Figure S2G.

Finally, the model was applied to assess the biological patina in the northern and southeastern pulpit areas before and after treatments, using the same preprocessing strategy and data reduction approach. This facilitated the evaluation and comparison of the biological patina and peperino stone within the PCA-LDA model across different treatments.

**Figure 9:**
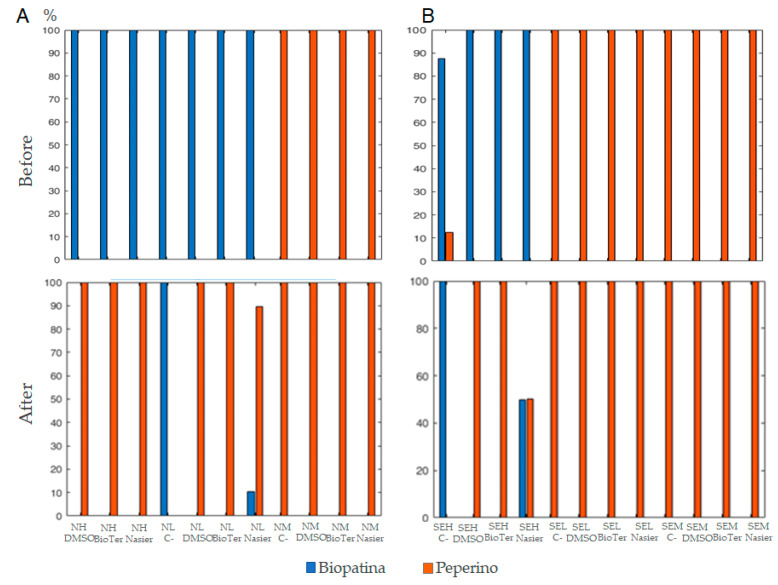
Predicted percentages of biopatina and peperino for each measurement area, as determined by PCA-DA, before (upper section) and after (lower section) treatments with DMSO-gel, BioTersus, and Nasier. Blue bars represent biopatinas and orange bars peperino. (**A**) North-facing side at three heights (i.e., high, low, mid), NH, NL, and NM, respectively. (**B**) Southeast-facing side at three heights, SH, SL, and SM, respectively. To improve reading of the results, of the two negative controls (C-) available at SEL and SEM, only one was shown.

By analyzing the obtained predictions, it is possible to highlight that the northern area shows a complex scenario with the presence of biological patinas in both the upper and lower sections (Figure 9A). In contrast, the central portion does not detect biological patina by the PVA-DA model. The predictions obtained from the analysis after treatment reveal a clear reduction of the biological patina in the upper section, with no significant differences observed among the three products applied (Figure 9A at the bottom: NH DMSO, NH BioTer, and NH Nasier). In the lower site of the pulpit, the high efficiency of the treatments based on DMSO and BioTersus^®^ is evident, while Nasier reduces the concentration of the biological patina without achieving total elimination (Figure 9A at the bottom: NL Nasier).

Differently, in the southeastern side, a biological patina was recorded in the upper area only (i.e., SEH, Figure 9B), while it is absent in the central (SEM) and lower site (SEL) (Figure 9B).

After treatments, significant differences in spectral emissions can be observed in the upper treated area compared to negative control (C−). Specifically, it can be noted that after treatment with DMSO and BioTersus, the spectral contribution of the biological patina is virtually absent, while in samples treated with Nasier, despite a significant reduction, the biological patina is still present (Figure 9B). In the control measurement (i.e., SEH A), no significant variations are observed, with the presence of the biological patina present before and after treatment, confirming that its disappearance in other areas is due to the applied treatments.

#### 3.3.2. LIF

LIF spectra were studied as they are and processed to gather fluorescence and distribution maps. The results obtained before treatments highlight differences in chlorophyll content among the areas at the three heights on the two different investigated sides.

**Figure 10:**
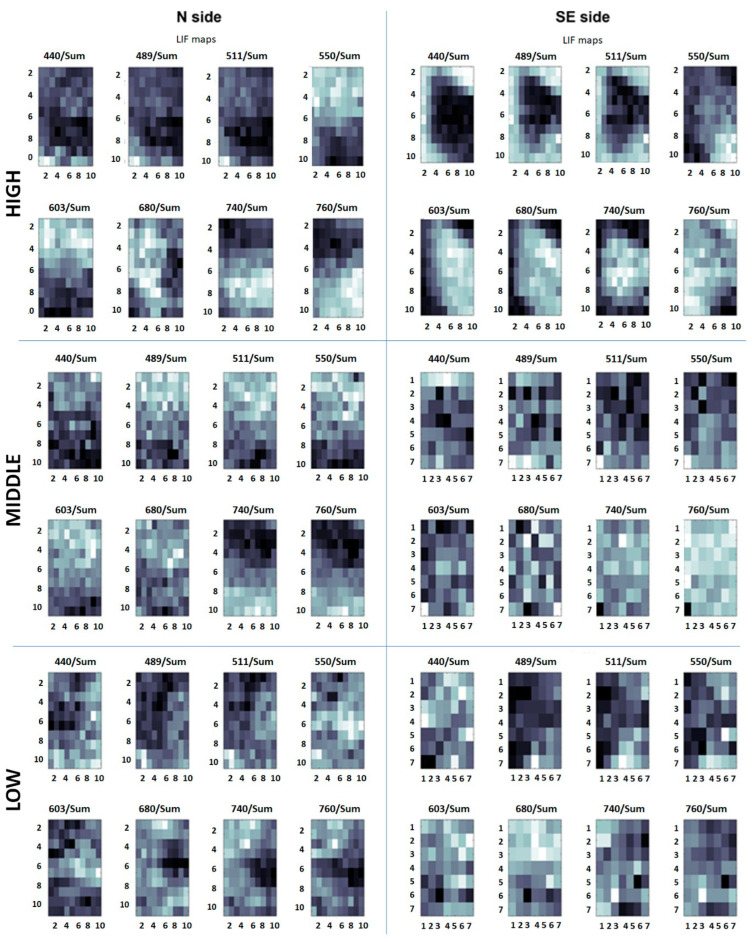
Fluorescence maps before treatments. Maps refer to one example area for each test site (NH, NM, NL on the left; SEH, SEM, SEL on the right). For each area, the fluorescence maps obtained by filtering at eight different wavelengths (440, 489, 511, 550, 603, 680, 740, and 780 nm) are shown.

In Figure 10, fluorescence maps at eight different wavelengths (440, 489, 511, 550, 603, 680, 740, and 780 nm) are presented and compared for three areas, for the high, middle, and low positions, respectively, for both the studied sides. Light colors correspond to higher fluorescence intensities; dark colors correspond to lower intensities at the measured wavelength. Maps clearly show the surface non-homogeneity for the high sites, in both the N and SE sides (i.e., NH and SEH). Additionally, they show an almost complete homogeneity at the middle and low elevation on the SE side, related to very noisy signals with a lack of well-defined fluorescence bands. An intermediate situation for the middle and low elevation on the N side can be observed.

**Figure 11:**
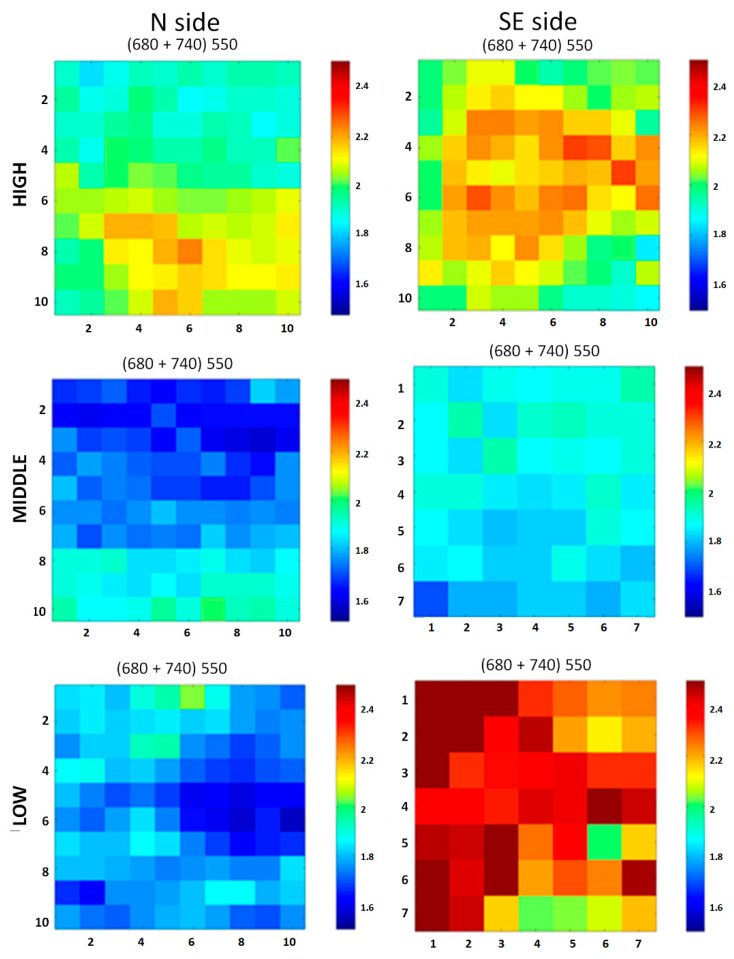
Chl *a*. distribution maps before treatments. Maps refer to one area at high, middle, low positions on the north (N, on the left) and southeast (SE, on the right) sides of the pulpit. SEM (SE, middle) and SEL (SE, low) sectors were smaller than the others, so only 7 pixel × 7 pixel squares were analyzed. In these last two areas, the low signal to noise ratio makes the maps not significative.

The maps in Figure 11 show the distribution of Chl *a* in the selected area at each elevation on both the SE and N sides; Chl *a* is abundant in SEH, quite abundant in NH, scarcely present in NM and NL, and not detectable in SEM and SEL; in these last two areas, the low signal to noise ratio makes the maps not significative.

Measurements repeated under the same experimental conditions after treatments did not reveal the presence of Chl *a* in any of the studied areas.

#### 3.3.3. HMI

To highlight the differences and similarities between the different treatments and their effectiveness in the removal of the surface biopatina, the algorithms of spectral similarity and reflectance comparison using PickViewer^®^ software were applied.

**Figure 12:**
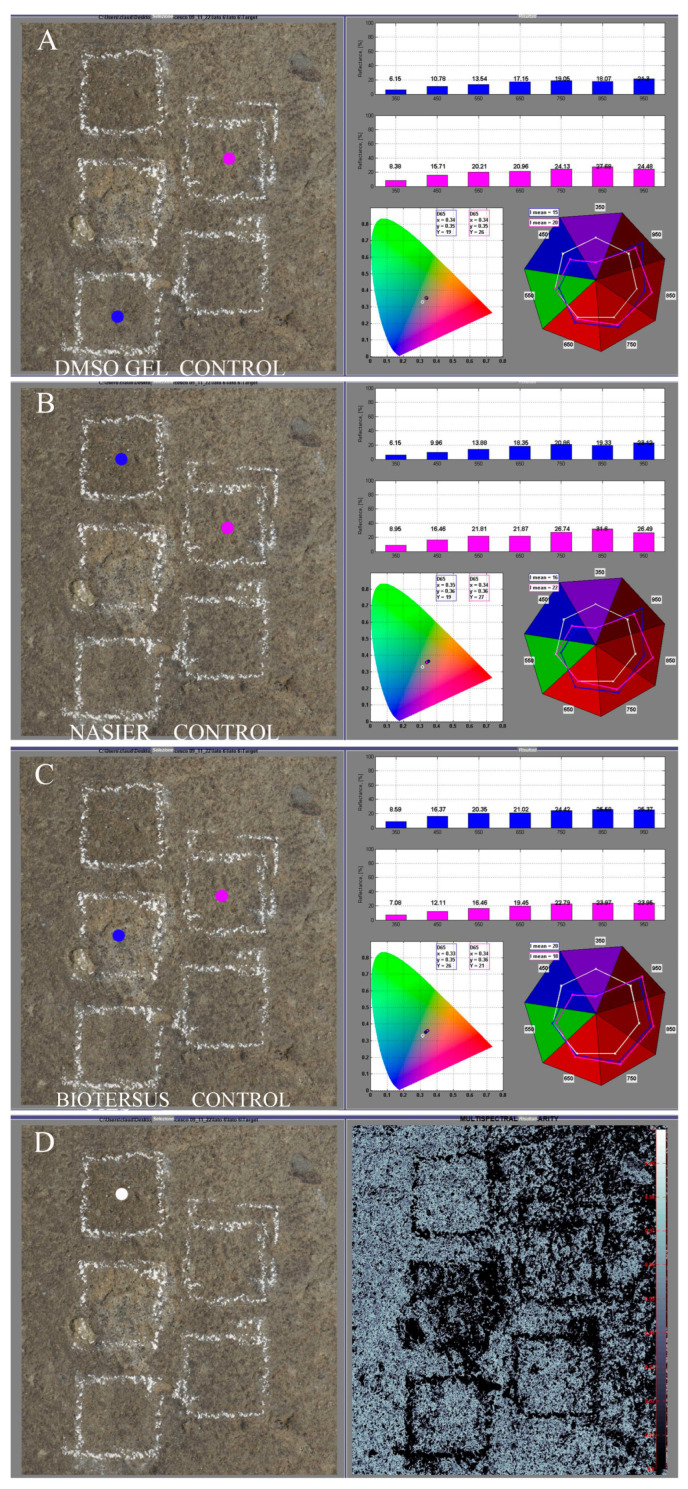
Graphical user interface of PickViewer^®^ software displaying the results of applying the reflectance comparison and spectral similarity tools to the middle area of the north side. (**A**) Comparison of the untreated area (control) vs. DMSO gel treatment; (**B**) comparison of the untreated area (control) vs. Nasier treatment; (**C**) comparison of the untreated area (control) vs. BioTersus treatment; (**D**) results of the spectral similarity algorithm applied to the point (white dot) visible in the RGB image (39 × 39 pixel); the white pixels in the right image indicate points with high similarity, while the black pixels show those with no similarity. Blue dots indicate the treated areas, and purple dots indicate the controls.

In the NM area, where the biopatina was thin but evident, some differences can be observed in the similarity maps and reflectance comparisons (Figure 12). The most evident change is associated with the BioTersus treatment, which seems to produce the best results in terms of biopatina removal. However, it should be noted that some peperino material detached as a consequence of the product removal (*Psyllium* pad).

**Figure 13:**
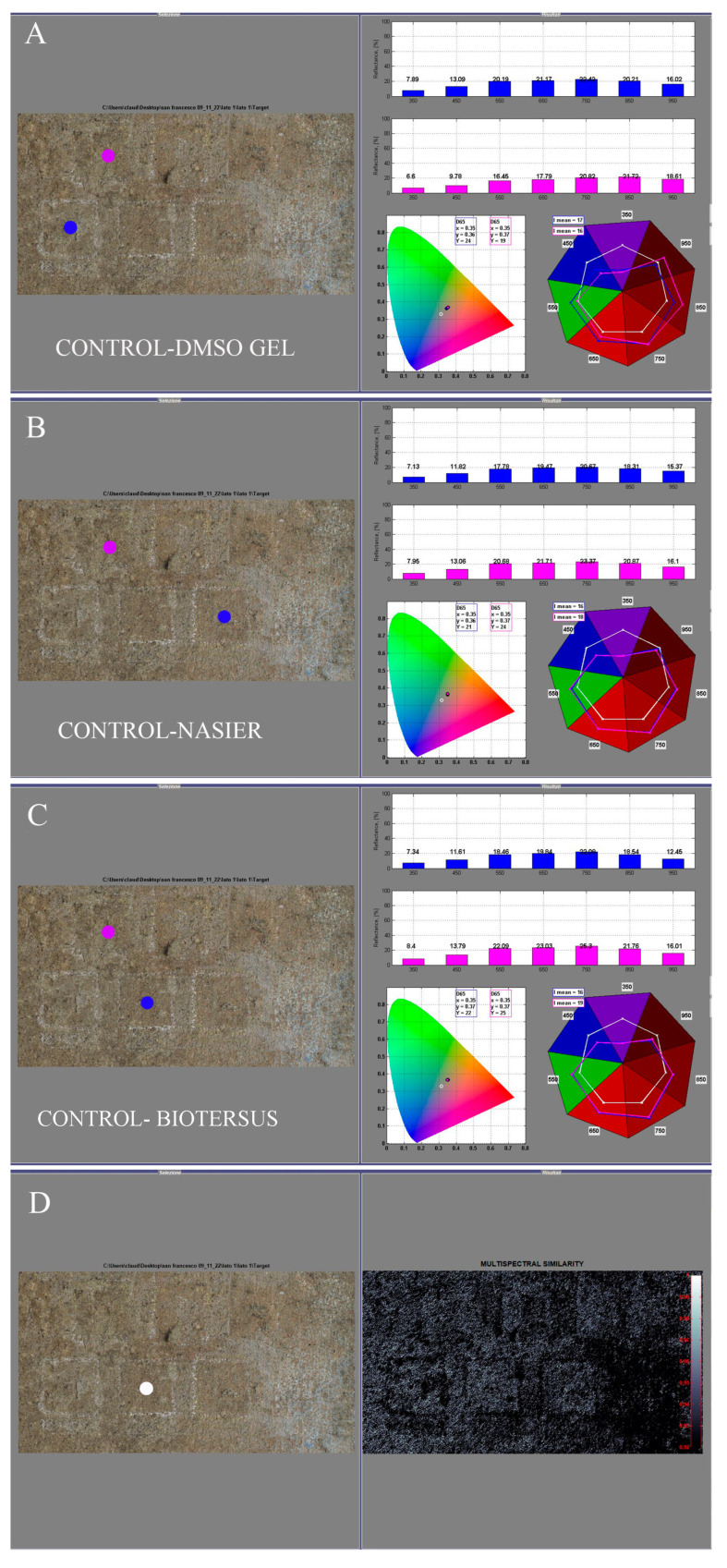
Graphical user interface of PickViewer^®^ software displaying the results of applying the reflectance comparison and spectral similarity tools to the middle area of the southeast side. (**A**) Comparison of the untreated area (control) vs. DMSO gel treatment; (**B**) comparison of the untreated area (control) vs. Nasier treatment; (**C**) comparison of the untreated area (control) vs. BioTersus treatment; (**D**) results of the spectral similarity algorithm applied to the point (white dot) visible in the RGB image (39 × 39 pixel): the white pixels in the right image indicate points with high similarity, and the black pixels show those with no similarity. Blue dots indicate the treated areas, and purple dots indicate the controls.

In the SEM area, the comparison in terms of spectral similarity and reflectance data shows that the treatments do not change the characteristics of the surface (Figure 13). This result agrees with absence of biopatina in the SEM side of the pulpit.

## 4. Discussion

Photosynthetic microorganisms are considered primary colonizers of stone surfaces, the earliest terrestrial niche [9,69], because of their phototrophism, low nutrient requirements [70], and the ability to produce the exopolysaccharides (EPSs) that promote the formation of microbial associations called biofilms (also known as biopatinas or subaerial biofilms (SABs)) [71,72]. Microbial communities exhibit a structure defined by physicochemical gradients, which shape microbial diversity, physiological activities, and their dynamics over time [73]. All the microbial mats recorded on the studied pulpit reflect this pattern, having water availability and sun exposure as the major drivers. The instrumental analysis precisely measured and confirmed what was observed during the initial objective examination, followed by direct microscope observation and, later, by culture-based analysis. Qualitative and quantitative differences between the north and southeast-exposed areas have been recorded. Biopatinas on the northern side of the pulpit were predominantly composed of phototrophic organisms. The biofilm thickness decreased in order from the upper (where rainwater accumulates on the pulpit platform) to the lower (affected by rising groundwater) and median part (distant from both water sources). In this last, the thin biopatina was dominated by BF while photoautotrophs were occasional. Unlike previous evidence [74], the presence of cyanobacterial species such as *Pleurocapsa* and algae like *Apatococcus* and *Lyngbya* on peperino stone cannot be solely attributed to calcareous substrates. Instead, they seem to be more influenced by surface roughness and porosity [74]. However, this assumption requires further confirmation through dedicated studies. Many of the photosynthetic microorganisms such as *Euglena*, *Oocystis*, *Apatococcus*, *Klebsormidium*, *Lyngbya*, *Leptolyngbya*, *Pleurocapsa*, *Chloroglea*, and *Calothrix* have also been found in fountains [75], in subterranean cultural heritage [68], and outdoor monuments [6]. This evidence underlines, on one hand, the preference of some microorganisms for stable water availability (e.g., *Oocystis* is generally associated with ponds) and, on the other hand, the strong adaptive traits of others such as *Apatococcus* [76], *Calothrix* [77], and *Klebsormidium* [78] showing a certain tolerance to desiccation. The tolerance to drought (and solar radiation) is also responsible for the distribution of BFs and lichens. Indeed, BFs were dominant in the NM, where they were associated with rare *Klebsormidium*, and in the SEH site with lichens. When environmental conditions reach their limits for sustaining life, such as in SEH (low water and high solar radiation) and SEL (high solar radiation and black oxalate crust), no biopatina was detected, either through biological analyses or instrumental analyses. While the findings for *Acarospora fuscata*, *Candelariella vitellina*, *Xanthoria parietina*, and *Scoliciosporum umbrinum* were previously recorded from Latium artworks [79,80], this study represents the first finding of black fungi from peperino stone. Some BFs, such as *K. petricola*, *K. marmoricola*, and *C. uncinatum*, were also found, mostly from marble surfaces at different latitudes [81]. The finding of *Scolecobasidium* sp. represents a novelty, as it has previously been found in environments such as caves and hydrocarbon-contaminated sites [82,83]. Additionally, several unidentified black fungal strains have also been isolated.

Conservation issues caused by biological growth can be addressed using both indirect and direct methods [84]. In the latter case, there has been a recent significant boost in the development of green and eco-friendly restoration/consolidation products and protocols to make cultural heritage conservation more sustainable [17,85,86]. This shift not only expands the methods available to restorers but, more importantly, safeguards the health of workers and the environment, potentially offering a safer alternative to traditional biocides. In some cases, the term ‘green’ has been associated with products of natural and especially vegetal origin, but this is not always the case. In a broader vision of sustainability, which includes safer health conditions for operators and the environment, measures should also be taken to reduce energy consumption, optimize resource use, minimize waste generation, and extend the service life of materials [87,88]. Despite the evidence that legitimizes the use of ‘green’ products, there are concerns suggesting the need for further testing on them [14,26]. In this light, we investigated the feasibility of using environmentally friendly products to remove biopatinas from peperino stone.

Considering the risks to which an artwork may be exposed due to excessive brushing or surface washing, it is of utmost importance to ensure the integrity of the artwork before starting and to calibrate the treatments to optimize results without compromising the artwork itself. The tests conducted on similar biopatinas, such as the one grown on the north side (NH) and the one recorded in the *ex situ* experiment (treated with both green and traditional products), demonstrated the effectiveness of all the tested protocols and highlighted the importance of having one or more effective options among the available methods. This allows the operator to choose the conditions that best suit the fieldwork, considering factors such as lower toxicity, ease of use, the size of the area to be treated, and, not least, management costs (e.g., materials, labor hours, etc.). From this perspective, the cost and the need for repeated applications might discourage the adoption of this approach or pose initial challenges for implementation. However, repeated applications are often necessary even with traditional biocidal treatments. In certain contexts, the ability to apply and remove treatments repeatedly can be an advantage, such as in fine-tuning the level of cleaning.

Differences in removal rates were recorded when the biopatina was more tenaciously attached to the stone substrate (SEH). The highest removal yield was achieved after two applications of DMSO-based gel, while BioTersus^®^ and Nasier left some lichen remains in slight and consistent amounts, respectively. No visible differences were recorded among the three methods applied in the other colonized sites, namely NL and NM. In this regard, the instrumental support becomes fundamental.

From an application perspective, the evidence collected in the field is essential for optimizing protocols, for example, the performances of the applied products and supporting materials. This may involve collaborating with suppliers or adjusting the application protocol to achieve the best possible results. Although temperatures were mild during the field experiments, dehydration emerged as the most critical factor to consider. For instance, when Nasier dried (due to stone porosity), it required intense mechanical action for removal. Similarly, the dehydration of *Psyllium* used as a support for BioTersus led to some peeling. However, the DMSO-based gel was less affected by dehydration due to its lower evaporation tendency [25]. To gain a broader understanding of these products, further studies on both colonized and uncolonized stones are necessary. These studies will assess the strengths to leverage and weaknesses to address from an operational perspective, particularly for potential use on larger surfaces.

Chlorophyll is the primary photosynthetic pigment in photoautotrophic organisms [89]. During the evolution process, different types of chlorophylls (i.e., a, b, c, d, and f) have differentiated to improve light-harvesting abilities and enhance adaptability to different environmental conditions. Chl *a* is universally distributed while Chl *b* can be found, for example, in Euglenophyta and Chlorophyta. Chl *c* can be found in groups such as Bacillariophyceae, Chrysophyceae, and Xanthophyceae, while the last two types are limited to a taxonomical group each. Indeed, Chl *d* can be found in Rhodophyta and Chl *f* in Cyanobacteria. Therefore, the presence or absence of a peculiar Chl is of taxonomic relevance [89,90].

Since autotrophic organisms are considered primary colonizers, the method for detecting biofilms using reflectance spectroradiometry and LIF primarily focuses on chlorophylls (while LIF mainly focuses on Chl *a*). In this context, the two methods showed a certain degree of comparability. Specifically, they provided full correspondence of results in four out of six sites (i.e., NH and NM (colonized), SEM, SEL (not colonized)) and partial correspondence in the remaining two (NL and SEH). In this regard, neither method was able to detect the colonization on NM, likely due to the rare presence of photoautotrophs and the dominance of BF. Similarly, fluorescence was not detected on the SEH BioTersus-treated tile, where the colonization was likely killed but not completely removed. Differences in detecting residual chlorophyll activity after Nasier treatment (i.e., NL and SEH) were recorded, with reflectance spectroradiometry showing higher sensitivity. This is most likely because LIF primarily focuses on Chl *a*. However, this comparison between the instrumental tools should not be seen as a definitive judgment that reflectance spectroradiometry is better than LIF but rather as an observation specifically limited to the case at hand, considering their various features and fields of application. The two instruments, in fact, work differently; reflectance spectroradiometry stays in contact, averaging the measured surface, while LIF operates at a one-meter distance (stand-off mode), mapping the whole surface in quasi real-time. The same applies to HMI, which, despite logistical challenges (e.g., difficulties in performing measurements at several meters above ground without the possibility of stabilizing the reference scale (NH)), provided essential information on the non-interference of the applied methods with the stone color. The field use of various non-invasive instruments applied to a material never studied before provided important insights for improving the equipment (e.g., sensitivity, detection range, ease of use, etc.), but further testing on other biofilms in the field is necessary. That is exactly as outlined in the COLLINE project.

## 5. Conclusions

This initial attempt to investigate biofilms and their removal from peperino stone monuments revealed that the DMSO-based gel provided the best removal yield for the biofilms examined. The results obtained with BioTersus were similar, although in the biofilm dominated by lichens, it was found to kill the biota without completely removing it. Nasier, in the applied conditions, proved effective on biofilms dominated by algae and cyanobacteria. None of these green treatments interfered with the color of the peperino stone. This could be of great interest due to the discoloration of peperino stone occasionally observed after treatment with traditional biocides.

All the instrumental tools provided important information about the distribution of biofilms before and after treatments. This experiment highlighted several aspects worth further investigation, both to improve the protocols for using eco-friendly products and to enhance the instrumentation performances.

## Data Availability

All data of this research is contained within the article and Supplementary Material.

## Supplementary Materials

The following supporting information can be downloaded at https://www.mdpi.com/article/10.3390/microorganisms13020375/s1. Figure S1: Post-treatment effects on tiles treated using DMSO-based gel, BioTersus, and Nasier, respectively; Figure S2: Preparatory steps for reflectance spectroradiometry; Table S1: Best BlastN match of the isolated fungal strains from the *ex situ* experiment (slab) and *in situ* experiment.
