# Supplementary material for: Biopatinas on Peperino Stone: Three Eco-Friendly Methods for Their Control and Multi-Technique Approach to Evaluate Their Efficacy
Source: Microorganisms. 2025 Feb 8;13(2):375. doi: 10.3390/microorganisms13020375 (PMC11858501; doi:10.3390/microorganisms13020375)
Supplement: Supplementary file 1 [file microorganisms-13-00375-s001.zip › microorganisms-3339393-supplementary.pdf]

microorganisms

MDPI

Supplementary Material

Biopatinas on Peperino stone: three eco-friendly methods for their control and multi-technique approach to evaluate their efficacy

Daniela Isola[1]*, Giuseppe Capobianco[2]*, Valery Tovazzi[1], Claudia Pelosi[1], Oriana Trotta[2], Silvia Serranti[2], Luca Lanteri[1], Laura Zucconi[3], and Valeria Spizzichino[4]

Table S1. Best BlastN match of the isolated fungal strains from the *ex situ* experiment (slab) and *in situ* experiment.

Sampled area	CCFEE	Fungal species	BLASTn match	% Identity	% Coverage	Accession nr
Slab	**10137**	*Coniosporium* sp.	*Coniosporium uncinatum* CCFEE 5820	97.09	99	PQ605752
NH	**10083**	*Setophaeosphaeria sp.*	*Setophaeosphaeria badalingensis isol.41*	99.28	99	PQ577887
NM	**10054**<br>**10057**	*Knufia marmoricola*	*K. marmoricola* CCFEE 5886	99.83	99	PQ577888
	**10056**	*Knufia petricola*	*Knufia petricola* CBS 600.93	99.83	99	PQ577889
NL	**10078**<br>**10152**	*Setophaeosphaeria* sp.<br>*Scolecobasidium* sp.	*Setophaeosphaeria badalingensis isol.41*<br>*Scolecobasidium robustum* CBS 112.97	99.28<br>96.52	100<br>80	PQ577890<br>PQ605753
SEH	**10050**<br>10053	*Constantinomyces* sp.	*Constantinomyces* sp. CCFEE 10001	99.6	99	PQ577891
	**10052**	Herpotrichiellaceae sp.	Melanized limestone ascomycete TRN68	99.23	97	PQ577892
	**10079**<br>10080	*Knufia marmoricola*	*Knufia marmoricola* CCFEE 6201	99.83	99	PQ577893
	**10081**	*Coniosporium uncinatum*	*C. uncinatum* CCFEE 10015	98.12	98	PQ577886
	**10051**	*Dothideomycetes* sp.	Dothideomycetes sp. CGMMCC 3.17080<br>Ascomycete CCFEE 5791	99.26<br>98.86	98<br>96	PQ577894
	**10049**	*Exophiala* sp.	*Exophiala bergeri* CBS 353.52	98.25	98	PQ577895

In situ experiment: NH: pulpit, north-exposed side, high position; NM: pulpit, north-exposed side, middle position; NL: pulpit, north-exposed side, low position; SEH: pulpit, south east exposed side, high position.

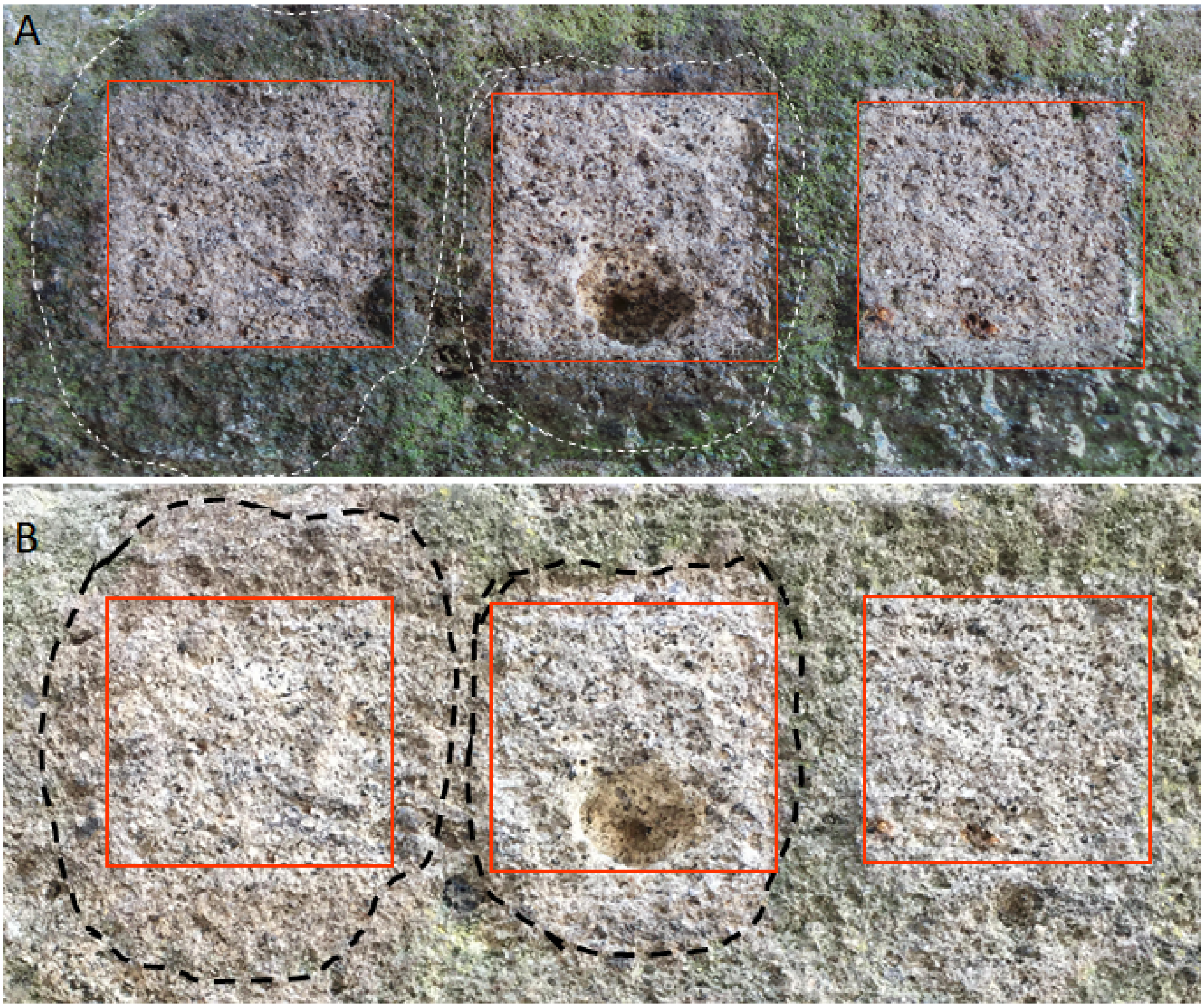

Figure S1. Post treatment effects on tiles treated using DMSO-based gel, BioTersus, and Nasier respectively. A) treated area at the end of the experiment; B) the same area 7 months later. Red squares indicate the treated areas, while the dashed lines (white in A, black in B) represent the wetted area during the brushing step and the area where the biopatina regressed, respectively. The two areas overlap perfectly.

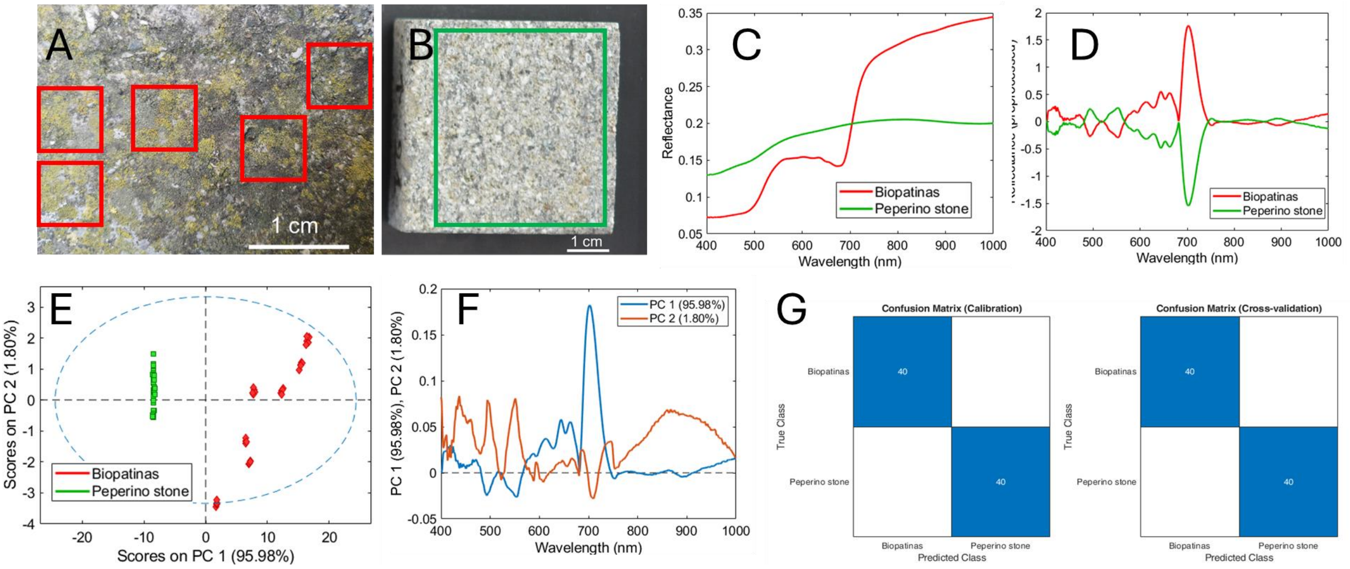

Figure S2. Preparatory steps for reflectance spectroradiometry. A, B) Reference used for sampling spectra: A) example of a region covered by biological patina, and B) reference peperino stone without biological patina; C, D) Reflectance measurements of biopatina and peperino stone within the 400-1000 nm range. C) Mean raw spectra; D) Pre-processed spectra using Detrend (linear), 1st Derivative (order: 2, window: 21 pt, incl only, tails: weighted), Absolute Value, Normalize (inf-Norm, Maximum = 1), and Mean Center. E, F) PCA reference plots for 'biopatina' and 'peperino stone': E) PCA score plot; F) PCA loading plot. G) Confusion matrix for calibration and cross-validation of the PCA-LDA model, distinguishing biopatinas and peperino stone.
